# Blood Pressure Changes During Aging and Menopause Among Forager‐Horticulturalists in the Bolivian Amazon

**DOI:** 10.1002/ajpa.70309

**Published:** 2026-07-21

**Authors:** M. J. Getz, T. Cao, J. E. Aronoff, C. L. Jenkins, S. Ghafoor, J. Vazquez, N. T. Appel, D. K. Cummings, P. L. Hooper, B. Beheim, K. H. Buetow, C. E. Finch, D. Eid Rodriguez, G. S. Thomas, R. C. Thompson, J. Stieglitz, M. Gurven, H. Kaplan, B. C. Trumble

**Affiliations:** ^1^ Center for Evolution and Medicine Arizona State University Tempe Arizona USA; ^2^ Institute of Human Origins Arizona State University Tempe Arizona USA; ^3^ School of Human Evolution and Social Change Arizona State University Tempe Arizona USA; ^4^ Integrative Anthropological Sciences University of California Santa Barbara Santa Barbara California USA; ^5^ School of Osteopathic Medicine in Arizona A.T. Still University Mesa Arizona USA; ^6^ Economic Science Institute Chapman University Orange California USA; ^7^ Department of Human Behavior Max Planck Institute for Evolutionary Anthropology Leipzig Germany; ^8^ Center for Economic and Social Research University of Southern California Los Angeles California USA; ^9^ Universidad San Simon Cochabamba Bolivia; ^10^ MemorialCare Heart & Vascular Institute MemorialCare Health System Fountain Valley California USA; ^11^ Division of Cardiology University of California Irvine Irvine California USA; ^12^ Saint Luke's Mid America Heart Institute University of Missouri‐Kansas City Kansas City Missouri USA; ^13^ Toulouse School of Economics Université Toulouse 1 Capitole Toulouse France

## Abstract

**Objectives:**

Cardiovascular disease (CVD) is the leading cause of death for women globally, and menopause is associated with elevated morbidity and mortality. The total impact of menopause on blood pressure remains inconclusive, with evidence largely based on data from industrialized populations. To address this gap, we examined associations between menopause, blood pressure, and hypertension among Tsimane forager‐horticulturalists and compared results against a nationally representative US sample to assess population‐level differences in blood pressure with menopause.

**Materials and Methods:**

We analyzed data from female and male Tsimane (*n*_individuals_ = 5,570; *n*_observations_ = 18,676; ages 15–92) and US National Health and Nutrition Examination Survey participants (NHANES; *n*_individuals_ = 6,861; ages 16–80). We applied linear regression models to assess age and population effects on systolic (SBP) and diastolic blood pressure (DBP) with menopause, logistic regression models for hypertension and stage 2 hypertension, and mediation analyses with estradiol.

**Results:**

SBP age trajectories were higher (*b* = 0.002, *p* < 0.001) and DBP trajectories lower (*b* = −0.004, *p* < 0.001) following menopause. Compared to US women, Tsimane women's SBP increased more quickly with age (*b* = 0.001, *p* = 0.021), while DBP decreased less dramatically (*b* = 0.003, *p* < 0.001). Postmenopause individuals had lower odds of hypertension with age compared to premenopausal women. Estradiol did not mediate blood pressure effects of menopause.

**Discussion:**

We found similar associations between menopause and blood pressure in both populations, despite widely differing CVD risk environments. However, Tsimane women showed slightly different pressure changes with age postmenopause. These results indicate blood pressure change postmenopause may be universal, but premenopause CVD risk environments may impact postmenopause CVD risk outcomes.

## 1 Introduction

The menopausal transition is associated with a variety of health challenges in industrialized populations, including elevated rates of cardiovascular disease (CVD), osteoporosis, and depression (Dosi et al. 2014; Santoro et al. 2015). CVD risk increases markedly postmenopause (Dosi et al. 2014; Uddenberg et al. 2024). While women typically experience lower incidence and better outcomes from certain CVD events earlier in life (Sun et al. 2021), women's CVD incidence surpasses men's around age 50 and CVD is the leading cause of death for women worldwide (Lansky et al. 2012; Vervoort et al. 2024; Cifkova et al. 2008; Coylewright et al. 2008). However, whether increased CVD risk stems directly from the hormonal and metabolic changes accompanying menopause or from aging itself remains unclear (Ryczkowska et al. 2022). High blood pressure is a commonly accepted risk factor for CVD (Oh and Cho 2020; Fuchs and Whelton 2020). In this article, “menopause” will refer to the suite of gradual biological shifts accompanying the cessation of menstruation, including reduced estrogens and progesterone, as well as increases in follicle‐stimulating hormone.

Lower estradiol levels after menopause may contribute to increased CVD risk. Estrogens play important and complex roles in the cardiovascular system, and their reduction is linked to increased inflammation, increased arterial stiffness, and decreased endothelial functioning (Knowlton and Lee 2012; Raj et al. 2023; Xing et al. 2009). Estradiol also influences the renin‐angiotensin‐aldosterone system, which plays key roles in sodium excretion and vasoconstriction, ultimately contributing to blood pressure regulation (Barris et al. 2023; Doshi and Agarwal 2013; Ishikawa et al. 2023). Declines in estrogens may impair these pathways, potentially increasing blood pressure.

Population‐level studies examining menopause and cardiovascular outcomes have reported mixed results, with some studies reporting increases in blood pressure. Research in the US (Gierach et al. 2006) has shown significant positive associations between menopause and systolic blood pressure, though other studies in Belgium (Staessen et al. 1997) and Italy (Casiglia et al. 2008) report no such associations after adjustment for age and body mass index (BMI). Disentangling the effects of menopause independent of age or BMI is a major challenge in this literature. Overall, work on menopause and its associations with blood pressure and hypertension have produced mixed results (Cifkova et al. 2008; Coylewright et al. 2008; Gierach et al. 2006; Oh et al. 2018). Although there is broad consensus that sex differences exist in blood pressure and hypertension risk with age, the role of menopause itself remains debated (Coylewright et al. 2008). Current evidence suggests blood pressure could vary by population with menopause, but methodological differences could explain this variation. Most research has been limited to industrialized populations, creating a gap in understanding the diversity of menopause and cardiovascular risk.

Existing studies on menopause and blood pressure have overwhelmingly been conducted with industrialized populations. However, differences in fertility schedules, hormone exposures, diet, and physical activity between industrialized environments and small‐scale subsistence societies may produce distinct cardiovascular outcomes. Studies among high‐fertility, physically active smaller‐scale populations may help improve current models that fail to capture broader heterogeneity of the menopause transition (Jasienska et al. 2017; Trumble et al. 2012). Documenting this variation is important, as it allows us to identify factors like diet, physical activity, and reproductive patterns that may contribute to differing CVD risk postmenopause. We worked with the Tsimane—Bolivian forager‐horticulturalists—to address this gap. The Tsimane have the lowest levels of coronary artery calcium (CAC) ever reported (Kaplan et al. 2017; Thompson et al. 2025) and low levels of atrial fibrillation and hypertension (Gurven et al. 2012, 2017; Kaplan et al. 2023; Rowan et al. 2021). These characteristics position them as a valuable comparator population to industrialized groups. We hypothesized that lower baseline CVD risk among Tsimane reduces the consequences of any potential increase in postmenopausal systolic blood pressure (SBP) and diastolic blood pressure (DBP), offering insight into the extent to which menopause effects are shaped by environmental context, including diet, physical activity, and fertility schedules. A comparative approach with both Tsimane and US women (see below) allows us to contrast postmenopausal outcomes in differing environments and widely differing lifestyles—one a high fertility, highly active population, and the other an industrialized, low fertility, relatively sedentary population—to better inform models of postmenopause CVD risk.

We collected cross‐sectional and longitudinal data from 2002 to 2022 on blood pressure and menopause status as a part of the Tsimane Health and Life History Project. To evaluate associations between menopause and cardiovascular risk across populations we ran analyses with this data and that of the 2017–2020 US National Health and Nutrition Examination Survey (NHANES n.d.).

Our primary goal was to assess whether menopause is associated with systolic or diastolic blood pressure or hypertension in a non‐industrial context, and to evaluate how these relationships compare to an industrialized setting. Overall, we expected the effect of menopause would be attenuated in the Tsimane compared to US women given their physically active subsistence strategies, low sodium diet, and lower lifetime cardiovascular risk exposure. Including male participants allowed us to examine blood pressure around the age of menopause and better isolate if results were menopause‐related (i.e., if women showed different trends than men of the same age), or age‐related. We aim to contribute cross‐population results on menopause, blood pressure, and hypertension by comparing a subsistence and an industrialized population, and to better inform models of the global heterogeneity of the menopause transition.

## 2 Materials and Methods

### 2.1 Sample

The Tsimane are an Indigenous Bolivian population of about 17,000 individuals, the majority of whom live in the Maniqui River basin, a tributary of the Amazon. The Tsimane live a highly physically active lifestyle. Fishing, foraging, hunting, and farming with hand tools are the most common subsistence activities (Gurven et al. 2017). Tsimane men and women have mean daily step counts of around 16,400 (SD = 5,300) and 16,280 (SD = 5,040), respectively, contrasting with the estimated 4,500 daily steps of US adults (Imms et al. 2025; Althoff et al. 2017). Communities have limited access to sanitation services, electricity, and pharmaceuticals (Dinkel et al. 2020; Gatz et al. 2023; Trumble et al. 2013, 2014). Tsimane women have 9.1 children on average throughout their lives (Gurven et al. 2016).

**FIGURE 1:**
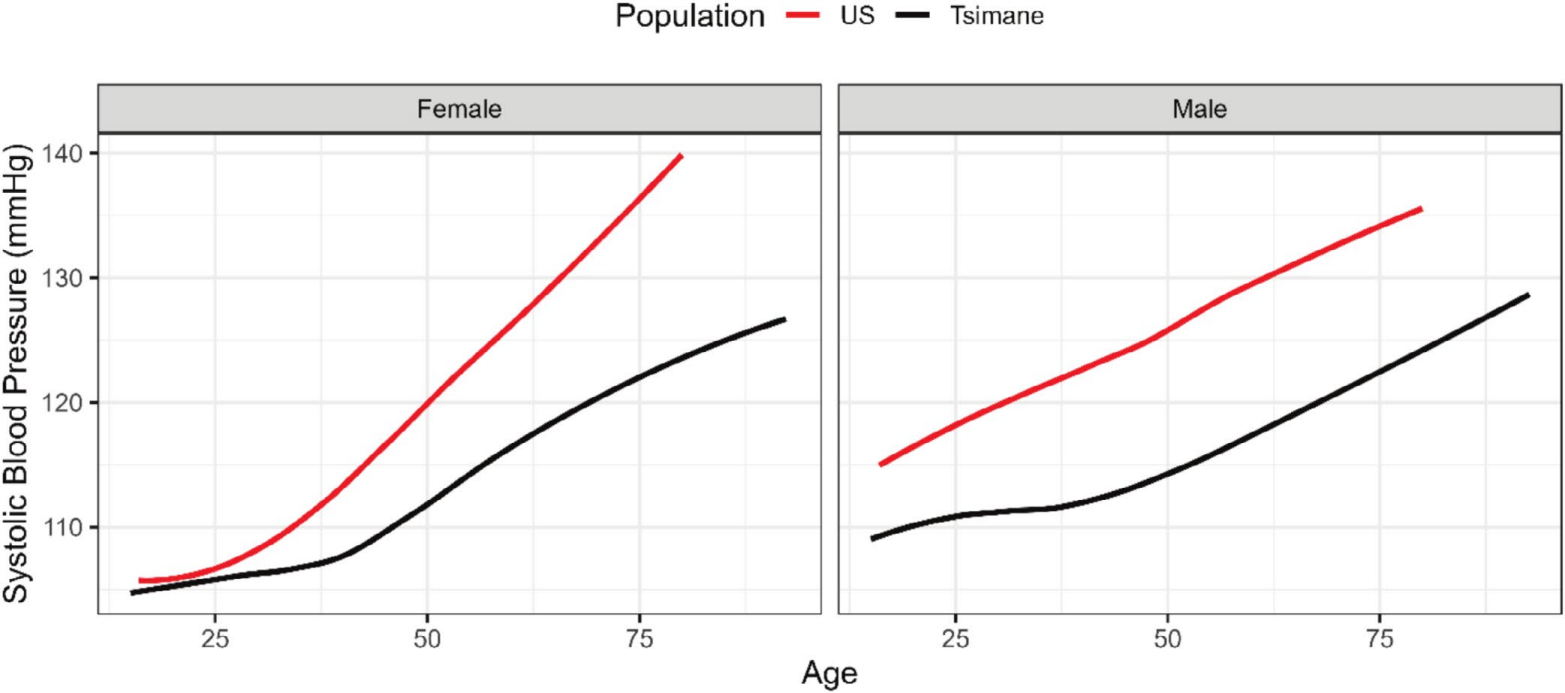
Systolic blood pressure (mmHg) with age (years) by population. Sexes show differing SBP trends, though difference is more pronounced in the US sample.

**FIGURE 2:**
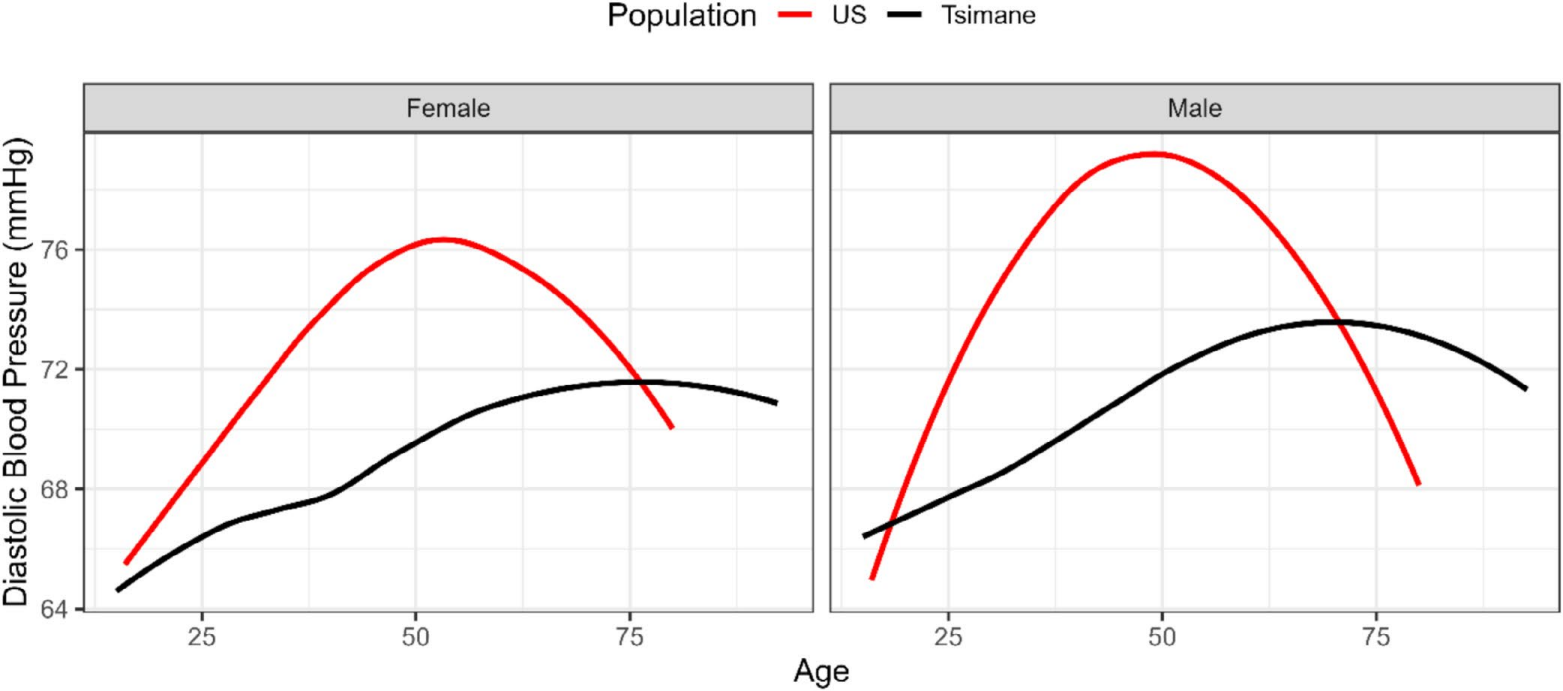
Diastolic blood pressure (mmHg) with age (years) by population. Sexes show similar DBP trends within each respective population.

Our sample was comprised of 9,989 blood pressure measurements from 2,821 Tsimane women ages 15–92 years. Our sampling strategy included all individuals over the age of 45 and age‐stratified random sampling of individuals aged 15–45. We also ran comparative analyses using data from the US NHANES, a cross‐sectional nationally representative survey of US adults. Our NHANES sample included 3489 women ages 16–80 from the 2017 to 2020 cycle, the most recent survey that included all variables of interest. Male Tsimane (*n* = 2,749, ages 15–92) and male NHANES participants (*n* = 3,372, ages 16–80) were included as comparator populations (Table 1, Figures 1 and 2).

**TABLE 1:** Characteristics of Tsimane and NHANES samples.

Characteristic	Tsimane women	Tsimane men	NHANES women	NHANES men
*N* individuals	2,821	2,749	3,489	3,372
*N* observations	10,025	8,651	3,489	3,372
Age range	15–92	15–92	16–80	16–80
Mean age (SD)	36.9 (16.4)	39.4 (16.4)	41.3 (17.7)	42.5 (18.8)
*N* observations premenopause	7,473	—	2,015	—
*N* observations postmenopause	2,552	—	1,214	—
Mean SBP (SD)	109.0 (13.7)	113.1 (13.2)	115.7 (17.6)	123.7 (15.9)
Mean DBP (SD)	67.7 (9.3)	69.9 (9.5)	72.0 (10.7)	74.2 (11.4)
Mean BMI (SD)	24.0 (3.7)	23.6 (2.8)	29.3 (8.2)	28.3 (6.5)
% Stage 2 hypertensive	5.2%	6.0%	8.6%	10.4%
% Systolic hypertension (SHT; prev.)	8.6%	9.8%	11.5%	16.6%
% Diastolic hypertension (DHT; prev.)	13.4%	17.6%	14.5%	19.5%
% Overall hypertension (HT; prev.)	16.8%	21.5%	18.5%	25.4%
Systolic incidence rate (SHT per 1,000 PY)	23.9	—	—	—
Diastolic incidence rate (DHT per 1,000 PY)	68.5	—	—	—
Overall incidence rate (HT per 1,000 PY)	76.6	—	—	—

*Note:* Values are mean (SD) or %. SBP/DBP in mmHg; BMI in kg/m^2^; PY = person‐years; Prev. age‐standardized to US 2000 Census.

### 2.2 Demographic Interviews

Demographic interviews were conducted in the Tsimane language with a bilingual (Spanish‐Tsimane) Tsimane research assistant. Years of birth were assigned and cross‐referenced using known ages from written records, photo comparisons of individuals with known ages, relative age rankings by multiple informants, and dated events (Gurven et al. 2007). Where possible, estimated ages were compared against Catholic mission birth records dating back to 1952. Each method produced a roughly independent age estimate, and the average of these estimates was used if all fell within a three‐year age range unless one method was considered superior to others.

### 2.3 Menopausal Status

Age at menopause was assessed using two methods: retrospective self‐reported menopausal status and, for premenopausal women, during medical visits approximately every 16 months in which women were queried by a Tsimane research assistant about the date of their last menstruation. This longitudinal data is a distinct study strength; women were also asked about the total number of live births they ever had, live births since their last health screening, total number of pregnancies (including miscarriages and other loss), and total number of pregnancies since their last screening, as well as the date of last menstruation. Among the Tsimane there is no taboo around discussing child death or fetal loss. However, early fetal loss is likely under‐detected and underreported (Gurven 2012). There is no specific word for menopause in Tsimane, and menopause status was back‐calculated from the date of a participant's last reported menstruation (Trumble et al. 2023). A subset of 624 women in our sample had detailed reproductive histories originally collected in 2002–2005 and updated through 2022. Forty‐one individuals self‐reported undergoing menopause before age 40 (~4% of our postmenopause sample). While some evidence suggests women in food‐limited environments may experience earlier menopause, an estimated less than 1% of women undergo menopause before the age of 40 in industrialized populations (MacMillan 2024; Elias et al. 2018; Scragg 1973). Given this low estimate, these individuals were removed from our postmenopause sample, as they may have been experiencing hypothalamic amenorrhea or another pathology. Individuals who reported menopause after age 65 (*n* = 40) were assigned menopause at age 50 (see below). Using this self‐report data, we ran a prospective analysis and used the mean age of women's cycling cessation as our menopause cutoff for participants without detailed reproductive histories. After cleaning, the mean age at menopause for women with self‐reported data was 47.5 (95% CI 47.2–47.9, see Figure S1 for the Tsimane distribution of age at menopause), and the 114 women over age 50 who lacked prospective data on age at menopause were assigned postmenopause status at age 50. Women assigned menopause at age 50 had a mean current age of 59.6 and were mainly older individuals who could not retroactively recall their exact age at menopause. In sensitivity analyses we removed these 114 individuals from our main models. Additionally, the same models were run and restricted to women with self‐report menopause status or those assigned menopause who were 55 years old or younger. For both analyses, our SBP age × population × menopause effect was no longer significant (see below), but our other results had no significant change. Tables S5 and S6 show results from additional analyses that included all women who self‐reported menopause before age 40. Again, coefficients and statistical significance remained largely unchanged.

### 2.4 Blood Pressure Collection Procedures

SBP and DBP were measured on the right arm with a Welch Allyn Tycos Aneroid 5090 sphygmomanometer (Welch Allyn, Skaneateles Falls, NY) and Littmann stethoscope (Littmann Stethoscopes, St. Paul, Minnesota) until 2013, and then with an Omron 3 Series automatic blood pressure cuff after 2013 (Omron, Kyoto, Japan). Participants were seated or supine for 20 min before measurements. After 2008, all blood pressure measurements were repeated after 30 min to confirm preliminary diagnoses (Gurven et al. 2012; Cao et al. 2025). We used the American Heart Association classification to define our hypertensive blood pressure categories (SBP ≥ 130 mmHg or DBP ≥ 80 mmHg; stage 2 hypertension was defined as SBP ≥ 140 or DBP ≥ 90 mmHg) (American Heart Association 2025).

### 2.5 Estradiol Biomarker Collection and Assay Procedures

Fasting morning blood (*n* = 834 specimens, 100% female) was collected and separated into serum before being frozen in liquid nitrogen and transferred to Arizona State University on dry ice before storage at −80°C. A commercial immunoassay was used to measure estradiol (Enzo Life Sciences, Farmingdale, New York).

### 2.6 Anthropometric Measure Collection Procedures

BMI was calculated using weight (kg) divided by standing height (m) squared. Standing height was measured without shoes to the nearest millimeter with a seca 213 mobile stadiometer (seca, Hamburg, Germany). Weight was collected using a TANITA BC‐1500 scale (TANITA, Tokyo, Japan).

### 2.7 NHANES Comparison

Our models included data from the 2017 to 2020 NHANES. NHANES data were cross‐sectional. Systolic and diastolic blood pressure variables were the mean of three blood pressure readings collected on the right arm for each individual after participants were seated for 5 min. For the 2017–2018 cycle, blood pressure was collected using a mercury sphygmomanometer (Bauman true gravity mercury wall model) with standard Bauman cuffs, as well as an HEM–907XL oscillatory device (Omron, Kyoto, Japan). After 2017–2018, the NHANES stopped collecting blood pressure using a mercury sphygmomanometer. NHANES phlebotomy was performed at a mobile examination center (MEC) during a morning, afternoon, or evening session. Fasting was not imposed for hormone collection. Samples were collected and processed at the MEC laboratory before being shipped on dry ice to US Centers for Disease Control contract laboratories for analysis. Estradiol analysis was performed using isotope‐dilution liquid chromatography–mass spectrometry.

NHANES question RHQ031 (“Have you had at least one menstrual period in the past 12 months?”) was recoded as a menopause variable, where NHANES responses of 1 (Yes) were considered premenopause, responses of 2 (No) were considered postmenopause, and responses of 3 (not sure), 7 (refused), and 9 (don't know) were considered not applicable. SDDSRVYR was used to identify each participant's two‐year survey cycle, and a year variable was defined as the midpoint of each participant's survey cycle. Individuals were excluded from analysis if they responded 1 (Yes) to question BPQ050A (“Are you now taking prescribed medicine for hypertensive blood pressure?”). Individuals on antihypertensive medications would have artificially low blood pressure, and thus were not included in the analysis (but see Tables S1 and S2 for additional sensitivity analyses including these individuals). No Tsimane are currently taking prescribed medicine for hypertensive blood pressure. As such, no Tsimane individuals were removed from the analysis.

### 2.8 Statistical Methods

All models were run in R version 4.4.3. ChatGPT‐5.2 and ‐5.4 were used to assist with coding. Model selection was based on Bayesian Information Criterion (BIC) and logged likelihood ratio tests (see Supporting Information). We performed a set of mixed‐effects linear regression models for log‐transformed systolic (*n*_observations_ = 12,615; *n*_individuals_ = 5,668) or diastolic blood pressure (*n*_observations_ = 12,612; *n*_individuals_ = 5,668) and included two interaction terms: mean centered BMI with population and a three‐way interaction term for mean centered age, menopause, and population. Models controlled for year of data collection and included individual ID as a random effect. Longitudinal data was provided by 71.2% of individuals in the Tsimane sample. 33% of postmenopausal Tsimane women provided longitudinal blood pressure data both pre‐ and postmenopause. We ran a second set of the same models including male participants to test if menopause effects on systolic (*n*_observations_ = 24,155; *n*_individuals_ = 11,512) and diastolic (*n*_observations_ = 24,147; *n*_individuals_ = 11,512) blood pressure were distinct from sex effects; these models included an interaction term for mean centered BMI with population, an interaction term for mean centered age, sex, and population, controlled for year of data collection, and included individual ID as a random effect.

#### 2.8.1 Logistic Regressions

We ran logistic regressions to model the probability of diastolic, systolic, and overall hypertension with menopause (*n*_hypertensio*n*_ = 12,868; *n*_systolic hypertension_ = 12,880; *n*_diastolic hypertension_ = 12,877). Regressions included one interaction term for population with mean centered and scaled BMI, one interaction term for mean centered and scaled age, menopause, and population, and controlled for year. BMI and age were mean centered and scaled to allow for model convergence. We ran a second logistic regression to model the probability of stage 2 hypertension after menopause among hypertensive individuals with the same interaction terms and controls (*n* = 12,602). All logistic regressions were also run with male participants (see Table S3).

Tsimane incidence rates of hypertension, or the total percentage of participants with hypertension in the sample, were calculated as a function of person‐time years in the sample. Hypertension prevalence rates, or the number of new hypertension cases per 1,000 person‐years in the study, were calculated for Tsimane and US samples in 10‐year age groups from age 15 to 85+. Tsimane prevalence was age‐standardized and weighted using the US 2000 standard population, and NHANES survey weights (WTMECPRP), strata (SDMVSTRA), and primary sampling units (SDMVPSU) were applied, then age‐standardized according to the US 2000 standard population. Both samples used the US 2000 standard population for comparability. NHANES hypertension prevalence was calculated using cross‐sectional data, while Tsimane prevalence rates were calculated with largely longitudinal data.

#### 2.8.2 Tsimane Mediation Analysis

We ran a set of mediation analyses with estradiol (measured from serum ELISAs [Enzo Life Sciences ADI‐901‐174]) as a mediator of menopause and log‐transformed systolic or diastolic blood pressure (*n*_observations_ = 834; *n*_individuals_ = 481). Due to positively skewed values, we log‐transformed estradiol. Mediation analyses accounted for changes within an individual over time and between individuals and controlled for mean centered age, year, BMI, and visit physician (to control for differences in manual blood pressure measurement). We included the estradiol ELISA kit and individual ID as random effects.

#### 2.8.3 NHANES Mediation Analysis

A mediation analysis was run for menopause and log‐transformed systolic or diastolic blood pressure with estradiol as a mediator, controlling for age and BMI (*n* = 2,399). To allow for comparability, we log‐transformed estradiol values.

Variance inflation factors (VIFs) were examined to check multicollinearity in main models. When we fit models without interaction terms, VIFs for all predictors were ≤ 2.8 (see Supporting Information).

### 2.9 Ethics

Informed consent was collected at three levels: from the individuals in the study, each participating community, and the governing body of the Tsimane (Gran Consejo Tsimane). All study protocols were approved by the Institutional Review Boards at the University of California Santa Barbara (#3‐21‐0652), the University of New Mexico (#07‐157), and the Universidad Mayor San Simón (Cochabamba, Bolivia).

## 3 Results

*Menopause was associated with blood pressure differences with age*: The slope of SBP was higher and slope of DBP lower with age postmenopause compared to premenopause after controlling for year and including interaction terms for population with BMI and population with age and menopause (Table 2). SBP (*b* = 0.002, *p* < 0.001) showed an increase of roughly 0.23 mmHg/year after menopause at the sample mean or increased approximately 0.2% per year after menopause. DBP (*b* = −0.004, *p* < 0.001) showed a roughly 0.4% per year decrease after menopause, or about 0.28 mmHg/year from the sample mean. After running models with identical controls but an interaction term for age and sex, rather than age and menopause, men did show significant differences in SBP (*b* = −0.002, *p* < 0.001) and DBP (*b* < −0.001, *p* = 0.002) with age, with slopes 0.23 mmHg/year and 0.07 mmHg/year lower than women at women's mean blood pressures (Table 3). SBP increased more rapidly with age after menopause among Tsimane compared to US women (*b* = 0.001, *p* = 0.021), while DBP decreased less rapidly with age after menopause among Tsimane women (*b* = 0.003, *p* < 0.001). Tsimane men showed significantly decelerated change in SBP (*b* = 0.001, *p* < 0.001) and DBP (*b* < 0.001, *p* = 0.003) slope with age compared to US men.

**TABLE 2:** US and Tsimane women mixed‐effects models.

Variable	SBP estimate (CI)	DBP estimate (CI)
Intercept	−2.407***	−3.615***
	(−3.568, −1.246)	(−4.978, −2.252)
Population: Tsimane vs. US	−0.011*	−0.016**
	(−0.021, −0.002)	(−0.027, −0.005)
	[%: −1.12 (−2.04, −0.19)]	[%: −1.61 (−2.67, −0.53)]
Age (per year)	0.003***	0.004***
	(0.003, 0.004)	(0.003, 0.004)
	[%/year: +0.34 (+0.29, +0.39)]	[%/year: +0.37 (+0.32, +0.43)]
BMI (kg/m^2^)	< 0.001***	0.005***
	(< 0.001, 0.001)	(0.004, 0.005)
	[% per kg/m^2^: +0.10 (+0.04, +0.15)]	[% per kg/m^2^: +0.46 (+0.40, +0.52)]
Menopause: Post vs. Pre	< 0.001	0.020*
	(−0.012, 0.014)	(0.004, 0.035)
	[%: +0.07 (−1.24, +1.40)]	[%: +1.99 (+0.43, +3.57)]
Year	0.004***	0.004***
	(0.003, 0.004)	(0.003, 0.005)
	[%: +0.35 (+0.30, +0.41)]	[%: +0.39 (+0.32, +0.46)]
Population × BMI	0.004***	< −0.001
	(0.003, 0.005)	(−0.002, < 0.001)
	[Δ % per kg/m^2^: +0.42 (+0.34, +0.51)]	[Δ % per kg/m^2^: −0.05 (−0.15, +0.05)]
Population × Age	−0.003***	−0.002***
	(−0.003, −0.002)	(−0.003, −0.002)
	[Δ %/year: −0.25 (−0.31, −0.19)]	[Δ %/year: −0.24 (−0.30, −0.17)]
Age × Menopause (Post vs. Pre)	0.002***	−0.004***
	(< 0.001, 0.002)	(−0.004, −0.003)
	[Δ %/year: +0.15 (+0.08, +0.22)]	[Δ %/year: −0.36 (−0.45, −0.28)]
Population × Menopause (Post vs. Pre)	−0.009	−0.013
	(−0.027, 0.010)	(−0.034, 0.008)
	[Δ %: −0.85 (−2.64, +0.98)]	[Δ %: −1.26 (−3.33, +0.85)]
Population × Age × Menopause (Post vs. Pre)	0.001*	0.003***
	(< 0.001, 0.002)	(0.002, 0.005)
	[Δ Δ %/year: +0.11 (+0.02, +0.21)]	[Δ Δ %/year: +0.34 (+0.23, +0.46)]
Sample size	*N* obs = 12,615; *n* indiv = 5,668	*N* obs = 12,612; *n* indiv = 5,668

*Note:* Estimate on log scale; CI = 95%.

* *p* < 0.05.

** *p* < 0.01.

*** *p* < 0.001. Bracketed lines show percent change; for age terms they are %/year; for interactions, the difference‐in‐slope (%/year).

**TABLE 3:** US and Tsimane population‐level mixed‐effects models.

Variable	SBP estimate (CI)	DBP estimate (CI)
Intercept	−0.932*	−4.224***
	(−1.766, −0.097)	(−5.213, −3.235)
Population: Tsimane vs. US	−0.012***	0.005
	(−0.018, −0.006)	(−0.002, 0.012)
	[%: −1.20 (−1.79, −0.62)]	[%: +0.49 (−0.21, +1.19)]
Age (per year)	0.004***	0.002***
	(0.004, 0.005)	(0.002, 0.002)
	[%/year: +0.44 (+0.42, +0.46)]	[%/year: +0.19 (+0.16, +0.21)]
BMI (kg/m^2^)	< 0.001***	0.005***
	(< 0.001, 0.001)	(0.005, 0.006)
	[% per kg/m^2^: +0.08 (+0.04, +0.12)]	[% per kg/m^2^: +0.55 (+0.50, +0.60)]
Sex: Male vs. Female	0.070***	0.033***
	(0.065, 0.076)	(0.027, 0.040)
	[%: +7.29 (+6.68, +7.90)]	[%: +3.38 (+2.70, +4.08)]
Year	0.003***	0.004***
	(0.002, 0.003)	(0.004, 0.005)
	[%: +0.28 (+0.24, +0.32)]	[%: +0.42 (+0.37, +0.47)]
Population × BMI	−0.002***	< −0.001**
	(−0.003, −0.002)	(< −0.001, < −0.001)
	[Δ %/kg/m^2^: −0.22 (−0.25, −0.20)]	[Δ %/kg/m^2^: −0.05 (−0.08, −0.02)]
Population × Age	0.004***	< −0.001*
	(0.004, 0.005)	(−0.002, < −0.001)
	[Δ % per year: +0.44 (+0.37, +0.51)]	[Δ % per year: −0.09 (−0.17, −0.01)]
Age × Sex	−0.002***	< −0.001**
	(−0.002, −0.002)	(< −0.001, < −0.001)
	[Δ %/year: −0.20 (−0.23, −0.17)]	[Δ %/year: −0.06 (−0.09, −0.02)]
Population × Sex	−0.035***	−0.002
	(−0.042, −0.028)	(−0.011, 0.006)
	[Δ %: −3.45 (−4.11, −2.77)]	[Δ %: −0.25 (−1.06, +0.57)]
Population × Age × Sex	0.001***	< 0.001**
	(< 0.001, 0.002)	(< 0.001, 0.001)
	[Δ Δ %/year: +0.11 (+0.07, +0.15)]	[Δ Δ %/year: +0.07 (+0.02, +0.12)]
Sample size	*N* obs = 24,155; *n* indiv = 11,512	*N* obs = 24,147; *n* indiv = 11,512

*Note:* Estimate on log scale; CI = 95%.

* *p* < 0.05.

** *p* < 0.01.

*** *p* < 0.001. Bracketed lines show percent change; for age terms they are %/year; for interactions, the difference‐in‐slope (%/year).

*Menopause was associated with lower odds of hypertension with age*: The odds of diastolic hypertension (OR = 0.37, *p* < 0.001), systolic hypertension (OR = 0.51, *p* < 0.001), and overall hypertension (OR = 0.62, *p* < 0.001) with age were significantly lower after menopause. Hypertensive individuals also had significantly lower odds of progressing to stage 2 hypertension after menopause (OR = 0.62, *p* = 0.028). Tsimane women had significantly greater odds of diastolic hypertension (OR = 3.26, *p* < 0.001), systolic hypertension (OR = 3.21, *p* < 0.001), overall hypertension (OR = 2.62, *p* < 0.001), and progressing to stage 2 hypertension (OR = 3.56, *p* < 0.001) after menopause than US women (Table 4).

**TABLE 4:** US and Tsimane women logistic regression models.

Term	Systolic HTN	Diastolic HTN	Any HTN	Stage 2 HTN
	OR (95% CI)	OR (95% CI)	OR (95% CI)	OR (95% CI)
Intercept	< 0.001***	< 0.001***	< 0.001***	< 0.001***
	(< 0.001, < 0.001)	(< 0.001, < 0.001)	(< 0.001, < 0.001)	(< 0.001, < 0.001)
Population: Tsimane vs. US	1.012	1.591***	1.640***	0.888
	(0.767, 1.336)	(1.301, 1.945)	(1.352, 1.990)	(0.642, 1.229)
BMI (per SD)	1.064	1.286***	1.238***	1.194***
	(0.992, 1.141)	(1.216, 1.360)	(1.172, 1.308)	(1.107, 1.289)
Age (per SD)	5.950***	2.952***	3.272***	4.461***
	(4.214, 8.402)	(2.386, 3.653)	(2.650, 4.039)	(3.119, 6.380)
Menopause: Post vs. Pre	1.326	1.291	1.060	0.991
	(0.927, 1.897)	(0.991, 1.683)	(0.807, 1.392)	(0.655, 1.501)
Year	1.076***	1.085***	1.085***	1.100***
	(1.056, 1.097)	(1.070, 1.100)	(1.071, 1.100)	(1.075, 1.125)
Population × BMI	1.650***	1.168**	1.252***	1.305***
	(1.462, 1.862)	(1.063, 1.284)	(1.142, 1.372)	(1.134, 1.503)
Age × Menopause (Post vs. Pre)	0.507***	0.368***	0.618***	0.619*
	(0.339, 0.758)	(0.281, 0.481)	(0.472, 0.810)	(0.403, 0.951)
Population × Age	0.370***	0.472***	0.444***	0.366***
	(0.245, 0.559)	(0.369, 0.605)	(0.348, 0.567)	(0.234, 0.573)
Population × Menopause (Post vs. Pre)	0.616*	0.643*	0.642*	0.660
	(0.381, 0.995)	(0.445, 0.931)	(0.445, 0.925)	(0.370, 1.177)
Population × Age × Menopause (Post vs. Pre)	3.212***	3.259***	2.620***	3.559***
	(1.943, 5.310)	(2.306, 4.606)	(1.865, 3.681)	(2.034, 6.229)
Sample size	*N* obs = 12,880	*N* obs = 12,877	*N* obs = 12,868	*N* obs = 12,602

*Note:* Cells show odds ratio (top) and 95% CI (bottom).

* *p* < 0.05.

** *p* < 0.01.

*** *p* < 0.001.

*Estradiol was not associated with blood pressure*: Estradiol was not associated with mediating differences in SBP or DBP among the Tsimane after menopause after controlling for age, year, BMI, and visit physician (Table 5). Among the US sample, estradiol did not significantly mediate a relationship between menopause and SBP or DBP after controlling for age and BMI (Table 6). However, estradiol analyses may be underpowered to detect a significant mediating effect of estradiol at *α* = 0.05, and results should be interpreted accordingly.

**TABLE 5:** Tsimane mediation analysis.

Term	Mediator (a‐path)	DBP outcome	SBP outcome
	Menopause → log(E2)	Estimate (CI)	Estimate (CI)
Menopause → log(E2) (a‐path)	−0.044		
	(−0.194, 0.107)		
	[%: −4.27 (−17.67, +11.32)]		
log(E2) within‐person (*b*_nx_)		< −0.001	< −0.001
		(−0.022, 0.020)	(−0.019, 0.019)
		[%: −0.09 (−2.19, +2.04)]	[%: −0.02 (−1.91, +1.91)]
log(E2) between‐person (*b*_ai_)		−0.011	−0.002
		(−0.029, 0.008)	(−0.020, 0.015)
		[%: −1.05 (−2.86, +0.80)]	[%: −0.25 (−1.95, +1.49)]
Menopause (direct *c*′)		0.001	< −0.001
		(−0.031, 0.034)	(−0.031, 0.030)
		[%: +0.14 (−3.05, +3.43)]	[%: −0.04 (−3.03, +3.04)]
Age (per year)		< 0.001	0.002***
		(< −0.001, 0.002)	(0.001, 0.003)
		[%/year: +0.08 (−0.04, +0.21)]	[%/year: +0.23 (+0.12, +0.35)]
Year (per year)		0.010	0.016**
		(< −0.001, 0.020)	(0.006, 0.027)
		[%/year: +0.97 (−0.06, +2.02)]	[%/year: +1.61 (+0.55, +2.69)]
BMI (kg/m^2^)		0.005***	0.007***
		(0.002, 0.007)	(0.005, 0.009)
		[% per kg/m^2^: +0.47 (+0.25, +0.70)]	[% per kg/m^2^: +0.68 (+0.46, +0.89)]
Visit physician		−0.138**	−0.225***
		(−0.237, −0.039)	(−0.316, −0.134)
		[%: −12.9 (−21.08, −3.87)]	[%: −20.1 (−27.08, −12.53)]
Sample size	*N* obs = 834; *n* indiv = 481	*N* obs = 827; *n* indiv = 481	*N* obs = 827; *n* indiv = 481

*Note:* All outcomes on log scale; values show estimate, 95% CI.

* *p* < 0.05.

** *p* < 0.01.

*** *p* < 0.001. Brackets show percent change; for age/year, they are % per year; for E2 terms, percent change in BP per 1‐unit change in log(E2).

**TABLE 6:** US mediation analysis.

Effect	DBP (mediate)	SBP (mediate)
	Estimate (CI)	Estimate (CI)
ACME	0.003	0.003
	(−0.003, 0.010)	(−0.002, 0.009)
	[%: +0.32 (−0.34, +0.98)]	[%: +0.33 (−0.23, +0.87)]
ADE	−0.005	< −0.001
	(−0.022, 0.012)	(−0.016, 0.016)
	[%: −0.50 (−2.22, +1.17)]	[%: −0.06 (−1.62, +1.57)]
Total effect	−0.002	0.003
	(−0.019, 0.015)	(−0.012, 0.017)
	[%: −0.18 (−1.86, +1.54)]	[%: +0.27 (−1.20, +1.74)]
Prop. mediated	−0.120	0.231
	(−11.294, 5.959)	(−6.093, 7.702)
Sample size	*N* obs = 2,399	*N* obs = 2,399

*Note:* Effects on log scale unless noted; ACME = indirect (mediated), ADE = direct, Total = ACME + ADE, Prop. Mediated = ACME/Total. Brackets show percent interpretation for log‐scale effects.

* *p* < 0.05.

** *p* < 0.01.

*** *p* < 0.001.

*Population differences*: US women's estradiol decreased 89% postmenopause, while Tsimane women's estradiol decreased 35% after menopause. See Table 1 for incidence and prevalence rates.

## 4 Discussion

Menopause was associated with increase in the age slope of SBP and decrease in the age slope of DBP among both Tsimane and US women. While some studies report associations between estradiol, menopause, and blood pressure, we found no such association for the Tsimane or US samples. Menopause was also associated with lower odds of hypertension with age in both samples.

Although men and women showed SBP and DBP differences with age, the menopause effect was either directionally opposite or larger in magnitude than the age‐sex interaction, indicating that menopause has an independent association with blood pressure beyond general sex differences.

US and Tsimane participants had lower odds of hypertension and stage 2 hypertension with age postmenopause. This may be due in part to epidemiological factors; while the odds of developing hypertension still increase postmenopause, more of the total women who will eventually develop hypertension may already be hypertensive before the menopause transition, leading to a deceleration with age (see Figure S2 for predicted odds of hypertension with age among the Tsimane pre‐ and postmenopause). Men showed similar patterns, with men having significantly lower odds of hypertension and systolic hypertension with age compared to women (see Table S3). In addition, Tsimane women's hypertension incidence was higher postmenopause compared to premenopause (Table S4). These results from male participants and increased incidence postmenopause further support that lower odds of hypertension with age may be an epidemiological artifact. However, significantly higher odds of developing all forms of hypertension with age postmenopause among Tsimane women may further support a role of menopause independent of age; menopause may be exerting an effect on blood pressure that counterbalances the Tsimane's protective environmental context.

Hypertension prevalence for both populations is more similar than we might expect (Table 1). However, while US SBP, DBP, and hypertension rates have remained largely stable over the past 15+ years, Tsimane hypertension rates have shown a marked increase over time (see Tsimane and US SBP, DBP, and hypertension prevalence by year in Figure S3). While Tsimane and US cardiovascular profiles remain distinct, these similarities may reflect increasing market integration and lifestyle shifts among the Tsimane.

US and Tsimane participants showed similar menopause/age associations, with Tsimane women showing more rapid increases in SBP with age postmenopause compared to US women and less pronounced decreases in DBP with age postmenopause. These findings suggest that while menopause‐associated changes in blood pressure with age may be a human universal, the magnitude may depend on population‐level CVD risk exposures.

Previous research on the associations between menopause and blood pressure shows widely varying results across studies and populations, which may be attributable to these population‐level differences in CVD risk environments, or the magnitude and duration of CVD risk factors (high access to processed foods, low levels of physical activity, etc.) an individual is exposed to throughout one's lifetime. While studies with participants from Italy, Japan, and the Czech Republic found no associations between blood pressure and menopause (Cifkova et al. 2008; Casiglia et al. 2008; Akahoshi et al. 1996), other work in Belgium did find significantly higher SBP postmenopause (Staessen et al. 1997), and studies in Italy and Taiwan found higher hypertension risk among postmenopausal women (Chen et al. 2014; Amigoni et al. 2000). Our results provide much‐needed context to this discussion by examining interactions between age, menopause, and population from two widely differing exposomes.

Our results indicate population‐level CVD risk environments may play key roles in shaping postmenopause CVD risk outcomes. DBP and SBP age trajectories varied between samples (Figures 1 and 2). These population‐level age trajectory differences are consistent with previous work that found 80‐year‐old Tsimane had the coronary health of US individuals in their mid‐50s, and research that found the Tsimane show blood pressure trajectories distinct from US patterns (Kaplan et al. 2017; Gurven et al. 2012). These population‐level differences throughout life may be due to major lifestyle and environment differences that may translate into different CVD risk outcomes with age. Tsimane CVD risk contrasts sharply with risk in the US, where prevalence of existing CVD across individuals age 30–79 is estimated at 9.6% and where prevalence of ischemic heart disease is expected to increase by 31.1%, heart failure by 33.0%, and stroke by 34.3% by 2060 (Faridi et al. 2025; Mohebi et al. 2022). The Tsimane have much higher physical activity levels than US adults and are less sedentary, with less than 10% of daylight hours spent on sedentary activities (Kaplan et al. 2017). They eat diets rich in fiber and polyunsaturated fatty acids and low in saturated fat and preservatives, smoke less, and show minimal development of chronic inflammation with age (inflammaging) (Rowan et al. 2021; Imms et al. 2025; Kraft et al. 2018; Aronoff et al. 2025).

These patterns reinforce the idea that population‐level cardiovascular aging is deeply shaped by environmental exposures. The LDL cumulative exposure hypothesis and related theories argue that both the magnitude and duration of risk exposure (e.g., elevated triglycerides, arterial pressure, and pulse pressure) drive disease progression (Ference et al. 2024; Domanski et al. 2023; Wang et al. 2017). Our results support these theories. When comparing SBP levels, the Tsimane are roughly 20–25 years behind the US—meaning a 75‐year‐old Tsimane woman has a SBP similar to a 50‐year‐old US woman (see Figure 1). The Tsimane peak in DBP is also roughly 25 years after the US peak, reflecting different cumulative exposure risk in blood pressure levels over the lifespan (Figure 2). These exposure and environment differences may lead to distinct changes in blood pressure with age postmenopause among the Tsimane, while overall associations with menopause remain similar across both populations.

In addition to population‐level CVD risk impacting postmenopause CVD blood pressure outcomes, varied associations between menopause and blood pressure could be partially attributed to the complex physiological role of estrogens in the cardiovascular system. Estrogens have a complex regulatory role in vascular elasticity, resulting in contradictory associations across studies. For example, estrogens are known to promote vasodilation and reduce cardiovascular inflammation (Prabhushankar et al. 2013). However, trials have also shown estrogens can act as a vasoconstrictor (Fardoun et al. 2020). We found no statistically significant associations between estradiol and blood pressure in either population. These results further highlight a need to understand the mechanistic role and effects of estradiol and other estrogens in the cardiovascular system.

While results are varied and estradiol is physiologically complex, existing research supports estradiol's protective role in preventing arterial stiffness and reducing risk of hypertension (Ashraf and Vongpatanasin 2006; Novella et al. 2012). Arterial stiffening plays a major role in isolated systolic hypertension, including increasing SBP (although it is unclear if this relationship is bidirectional) (Franklin 2008; AlGhatrif and Lakatta 2015). However, our results found no association between estradiol and blood pressure among Tsimane or US women. One possible explanation could be that differences in vascular calcification, rather than estrogens, may be a more influential mechanism in postmenopause arterial stiffening and cardiovascular outcomes.

CAC is a measure of atherosclerosis (Stary et al. 1995). Vascular calcification is associated with a variety of CVD risk markers, including cholesterol and other lipids (Sage et al. 2010; Demer 2001). Oxidized low‐density lipoprotein (LDL) cholesterol has been linked to atherosclerotic calcification, and in older US women, high‐density lipoprotein (HDL) cholesterol appears to lose some of its protective effects against calcification in the coronary arteries and aorta (Witztum and Steinberg 1991; Woodard et al. 2011; Kuller et al. 1999). In addition to a reversal of HDL's protective effects, lower levels of estrogens have been shown to increase oxidative stress levels, potentially increasing the oxidation of low‐density lipoprotein cholesterol and contributing to arterial calcification (Doshi and Agarwal 2013). While further work is needed, one study found a positive association between menopause and CAC among Brazilian women (Fonseca et al. 2022). Previous work with the Tsimane found positive associations between menopause and a variety of lipids—including low‐density lipoprotein cholesterol. While the Tsimane associations had smaller effect sizes, they were similar to patterns seen in US/UK populations (Getz et al. 2025). These results support a potential link between menopause and vascular calcification, especially in environments with higher CVD risk (more access to processed foods, less physical activity, etc.). Future work is needed to further examine the associations between vascular calcification and menopause status, especially in nonindustrial populations.

The Tsimane show a more rapid increase in SBP and a decelerated decline in DBP with age postmenopause compared to US women, as well as higher risk of systolic, diastolic, stage 2, and overall hypertension with age postmenopause. While we are unable to assess causality, hormone differences from high fertility and long periods of lactation among the Tsimane may lead to arterial differences between Tsimane and US women. For example, estradiol levels increase substantially during pregnancy and decrease during breastfeeding (Dukic et al. 2024; Battin et al. 1985). Differences in lifetime estradiol exposure may contribute to population‐level variation in CVD risk. Compared with US women, who generally have fewer births and greater access to hormonal contraception that reduces estradiol fluctuations across the menstrual cycle, Tsimane women likely experience a distinct hormonal profile over the life course. Although this study identified substantial declines in estradiol from pre‐ to postmenopause in both populations (89% in US women and 35% in Tsimane women), estradiol was not associated with postmenopausal blood pressure in the Tsimane. Future work should clarify estradiol's role postmenopause and the mechanisms linking it to cardiovascular health. Additionally, low lifetime CVD risk may amplify sensitivity to cardiovascular changes with menopause among Tsimane women. Future work should further examine factors underlying these population‐level differences.

These findings suggest differences in blood pressure with age after menopause may be a human universal, but that the extent of these differences varies by population and are impacted by premenopause CVD risk environments, or the magnitude and duration of CVD risk factors (high access to processed foods, low levels of physical activity, etc.) an individual is exposed to premenopause. This aligns with previous work that found Tsimane and US/UK populations both showed lipid changes after menopause, but that the extent of these changes seemed to be influenced by CVD risk environment (Getz et al. 2025). Together, these results support cumulative exposure models of CVD, indicating that duration and magnitude of risk exposure matter for cardiovascular outcomes. Additionally, results suggest arterial calcification, rather than arterial stiffening, may be a prominent driver of cardiovascular risk after menopause.

### 4.1 Limitations

US data did not include subgroups for estrogen replacement therapy, which has been associated with lower all‐cause mortality and CVD (Hodis and Mack 2022). We were not able to account for genetic differences or gene–environment interactions that may influence the differences in results between groups. Although the American Heart Association has a defined elevated category for SBP, an elevated analysis was omitted as there is no equivalent category for DBP. Neither Tsimane nor NHANES data included menstrual cycle day. NHANES data were cross‐sectional, and so we are unable to calculate incidence rates. Differing blood pressure collection methods between NHANES and THLHP (mercury sphygmomanometer until 2017–2018 and Omron automatic device in NHANES versus mercury sphygmomanometer until 2013 and Omron automatic device in THLHP) cannot be accounted for. Blood pressure was collected seated or supine among the Tsimane. However, there is no difference in seated versus supine collection for SBP and very little for DBP, and it is unlikely this modified the results (Netea et al. 1998). Two different methods were used to measure estradiol in the two samples—ELISA in one and chromatography–mass spectrometry in the other—so direct comparisons were not possible. Estradiol was therefore analyzed separately in each sample.

## 5 Conclusion

We found associations between menopause and blood pressure increases with age among US and Tsimane participants, and while associations were similar between both groups, Tsimane women showed pronounced differences postmenopause compared to US women. The Tsimane and US women in this study live in very different environments in terms of diet and physical activity; the Tsimane have extremely low levels of cardiac morbidity and the lowest levels of CAC ever reported, while the US is an industrialized, high‐CVD risk environment. Our results suggest that blood pressure changes postmenopause may be a human universal, but that CVD risk environments may exacerbate or attenuate postmenopause CVD outcomes.

In sum, our results support the interpretation that menopause‐related changes in blood pressure with age may be a universal feature of aging, but may also be context‐dependent and shaped by specific ecological and lifestyle environments. This cross‐cultural evidence provides important insight into global variation in cardiovascular aging and highlights the need for models that account for environmental and population‐level heterogeneity in menopause outcomes.

## Author Contributions

**D. K. Cummings:** data curation, writing – review and editing. **R. C. Thompson:** conceptualization, methodology, funding acquisition, project administration, writing – review and editing. **J. Vazquez:** writing – review and editing. **J. E. Aronoff:** writing – review and editing, data curation. **M. J. Getz:** conceptualization, methodology, formal analysis, validation, investigation, visualization, writing – original draft, writing – review and editing. **D. Eid Rodriguez:** writing – review and editing, data curation. **B. Beheim:** data curation, writing – review and editing. **S. Ghafoor:** software. **G. S. Thomas:** funding acquisition, project administration, conceptualization, methodology, writing – review and editing. **H. Kaplan:** conceptualization, methodology, investigation, validation, formal analysis, supervision, funding acquisition, project administration, writing – review and editing. **N. T. Appel:** writing – review and editing. **P. L. Hooper:** data curation, writing – review and editing. **T. Cao:** writing – review and editing, formal analysis, data curation. **C. L. Jenkins:** writing – review and editing. **K. H. Buetow:** writing – review and editing. **J. Stieglitz:** funding acquisition, project administration, writing – review and editing. **B. C. Trumble:** conceptualization, methodology, investigation, validation, formal analysis, supervision, funding acquisition, project administration, writing – review and editing. **M. Gurven:** project administration, writing – review and editing, funding acquisition, conceptualization, formal analysis, methodology, investigation, validation, supervision. **C. E. Finch:** writing – review and editing.

## Funding

This work was supported by the National Institute on Aging within the National Institutes of Health (R01AG054442), National Science Foundation (1748282), and French National Research Agency under the Investments for the Future (Investissements d'Avenir) program (ANR‐17‐EURE0010). This material is based upon work supported by the National Science Foundation Graduate Research Fellowship Program under Grant No. 2233001. Any opinions, findings, and conclusions or recommendations expressed in this material are those of the author(s) and do not necessarily reflect the views of the National Science Foundation.

## Ethics Statement

Informed consent was collected at three levels: from the individuals in the study, each participating community, and the governing body of the Tsimane (Gran Consejo Tsimane). All study protocols were approved by the Institutional Review Boards at the University of California Santa Barbara (#3‐21‐0652), the University of New Mexico (#07‐157), and the Universidad Mayor San Simón (Cochabamba, Bolivia).

## Conflicts of Interest

The authors declare no conflicts of interest.

## Supporting information


**Table S1:** US and Tsimane women linear regression models including NHANES participants on anti‐hypertensive medication.
**Table S2:** US and Tsimane women logistic regression models including NHANES participants on anti‐hypertensive medication.
**Table S3:** US and Tsimane men logistic regression models.
**Table S4:** Tsimane pre‐ versus postmenopause hypertension incidence rates.
**Table S5:** Linear and logistic regression results including Tsimane who self‐reported menopause before age 40.
**Table S6:** Estradiol sensitivity results including Tsimane who self‐reported menopause before age 40.
**Figure S1:** Distribution of Tsimane age at menopause.
**Figure S2:** Tsimane odds of hypertension pre‐ versus postmenopause.
**Figure S3:** Tsimane and US SBP, DBP, and hypertension prevalence by year.

## Data Availability

Individual‐level data are stored in the THLHP Data Repository and are available through restricted access for ethical reasons. THLHP's highest priority is the safeguarding of human subjects and minimization of risk to study participants. The THLHP adheres to the “CARE Principles for Indigenous Data Governance” (Collective Benefit, Authority to Control, Responsibility, and Ethics), which assure that the Tsimane (i) have sovereignty over how data are shared, (ii) are the primary gatekeepers determining ethical use, (iii) are actively engaged in data generation, and (iv) derive benefit from data generated and shared for use whenever possible. The THLHP is also committed to the “FAIR Guiding Principles for scientific data management and stewardship” (Findable, Accessible, Interoperable, Reusable). Requests for individual‐level data should take the form of an application that details the uses of the data, the research questions to be addressed, procedures that will be used for data security and individual privacy, potential benefits to the study communities, and procedures for assessing and minimizing stigmatizing interpretations of the research results (see the following webpage for links to the data sharing policy and data request forms: https://tsimane.anth.ucsb.edu/data.html). Requests for individual‐level data will require institutional IRB approval (even if exempt) and will be reviewed by an Advisory Council composed of Tsimane community leaders, community members, Bolivian scientists, and the THLHP leadership. The study authors and the THLHP leadership are committed to open science and are available to assist interested investigators in preparing data access requests.
