# Supplementary material for: Blood Pressure Changes During Aging and Menopause Among Forager‐Horticulturalists in the Bolivian Amazon
Source: Am J Biol Anthropol. 2026 Jul 21;190(3):e70309. doi: 10.1002/ajpa.70309 (PMC13389348; doi:10.1002/ajpa.70309)
Supplement: Supplementary file 1 — Table S1: US and Tsimane women linear regression models including NHANES participants on anti‐hypertensive medication. Table S2: US and Tsimane women logistic regression models including NHANES participants on anti‐hypertensive medication. Table S3: US and Tsimane men logistic regression models. Table S4: Tsimane pre‐ versus postmenopause hypertension incidence rates. Table S5: Linear and logistic regression results including Tsimane who self‐reported menopause before age 40. Table S6: Estradiol sensitivity results including Tsimane who self‐reported menopause before age 40. Figure S1: Distribution of Tsimane age at menopause. Figure S2: Tsimane odds of hypertension pre‐ versus postmenopause. Figure S3: Tsimane and US SBP, DBP, and hypertension prevalence by year. [file AJPA-190-e70309-s001.docx]

SUPPLEMENTAL MATERIALS AND METHODS

**Statistical Methods**

*Logistic Regressions.* In addition to mixed-sample regressions, we ran mixed-effects logistic regressions to model the probability of diastolic hypertension, systolic hypertension, and overall hypertension with menopause separately for each population. Tsimane models controlled for age, BMI, year of data collection, z-scored age^2^ (for systolic and overall hypertension), and visit physician, and age, BMI, and year of data collection were z-scored to allow for better model convergence. U.S. models controlled for age, z-scored age^2^ (for systolic and overall hypertension), and BMI. We also ran a logistic regression for modeling the probability of systolic and/or diastolic stage 2 hypertension after menopause in the Tsimane alone. These models controlled for z-scored age, BMI, year of data collection, and z-scored age^2^ (for systolic and overall hypertension). The models that included both populations (reported in the main manuscript) were better model fits according to BIC and LRT.

All main logistic regression models were run with male participants to compare sex versus menopause effects; these models included an interaction term for mean centered and scaled age, sex, and population as well as a mean centered and scaled BMI and population interaction term and a control for year (n_hypertension_=24,797; n_diastolic hypertension_=24,811; n_systolic hypertension_=24,819; n_stage 2_=24,132). Similar to models with female participants, men had significantly lower logged odds of systolic hypertension, diastolic hypertension, and overall hypertension with age compared to female participants, and hypertensive individuals had significantly lower logged odds of stage 2 hypertension with age compared to female participants (see SI Table 1).

*VIFs.* VIFs for full models that included interaction terms ranged from 1.6 to 33.9. This mathematical inflation of VIFs is expected given predictors were included in multiple interaction terms. Year and visit physician GVIFs exceeded 25.0 in Tsimane mediation models; physicians often worked year-long contracts, making this collinearity unavoidable. Models were run with visit physician, without visit physician, and with visit physician as a random effect, and including visit physician did not significantly change the results. All other variables in the Tsimane mediation model were under 2.0.

*Model Selection.* Main models were run with a three-way interaction between menopause, population, and age and compared against a model with a two-way interaction between menopause and age with no population variable. While BIC was slightly higher, AIC was slightly lower and LRT suggested the three-way interaction model was a borderline significantly better fit. Adding the three-way interaction did not substantively change the model outcome, and was selected as the final model.

*Menopause Age.* A sensitivity analysis was run that included all 41 Tsimane individuals who self-reported undergoing menopause before age 40. Our population x menopause interaction term in the DBP and SBP linear regressions and in all hypertension logistic regressions showed the largest changes in coefficients in the sensitivity analysis, with coefficient changes of 38.5% and -11.1% for linear models and 30.4% (DHT), 11.8% (stage 2), 34.5% (overall hypertension), and -37.7% (SHT) from the original model (see SI Table 5). Population x menopause in our diastolic hypertension and overall hypertension models was no longer significant. However, population x menopause was not statistically significant in all other original models and remained non-significant in our sensitivity analyses. Similarly, our mediation analyses including Tsimane women with self-reported menopause before age 40 (see SI Table 6) showed a -50.0% coefficient change for between-person estradiol for SBP and a 9.1% coefficient change for between-person estradiol for DBP but showed no change in significance.

**SI Table 1: U.S. and Tsimane Women Linear Regression Models Including NHANES Participants on Anti-Hypertensive Medication**

**
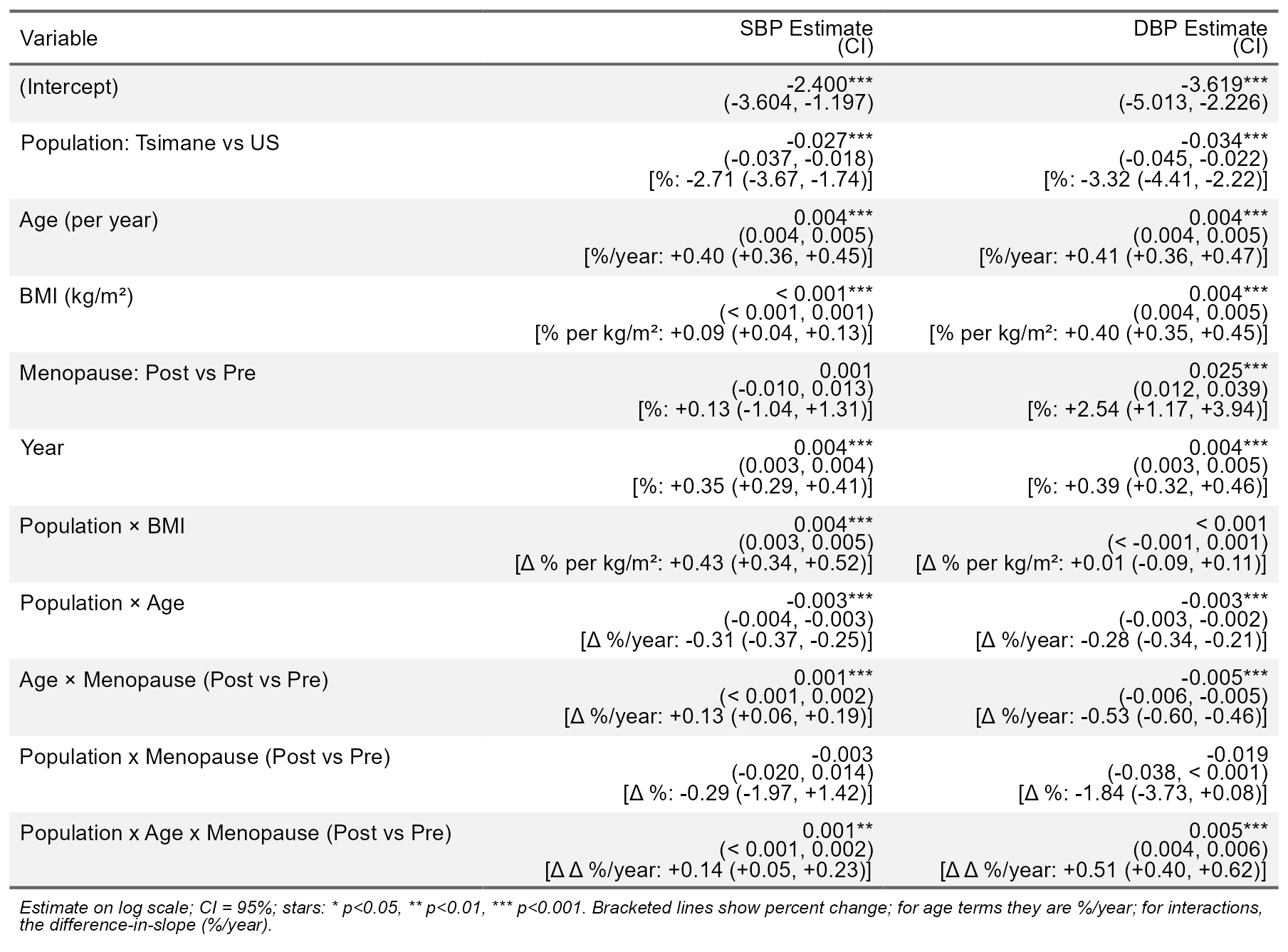
**

**SI Table 2: U.S. and Tsimane Women Logistic Regression Models Including NHANES Participants on Anti-Hypertensive Medication**

**
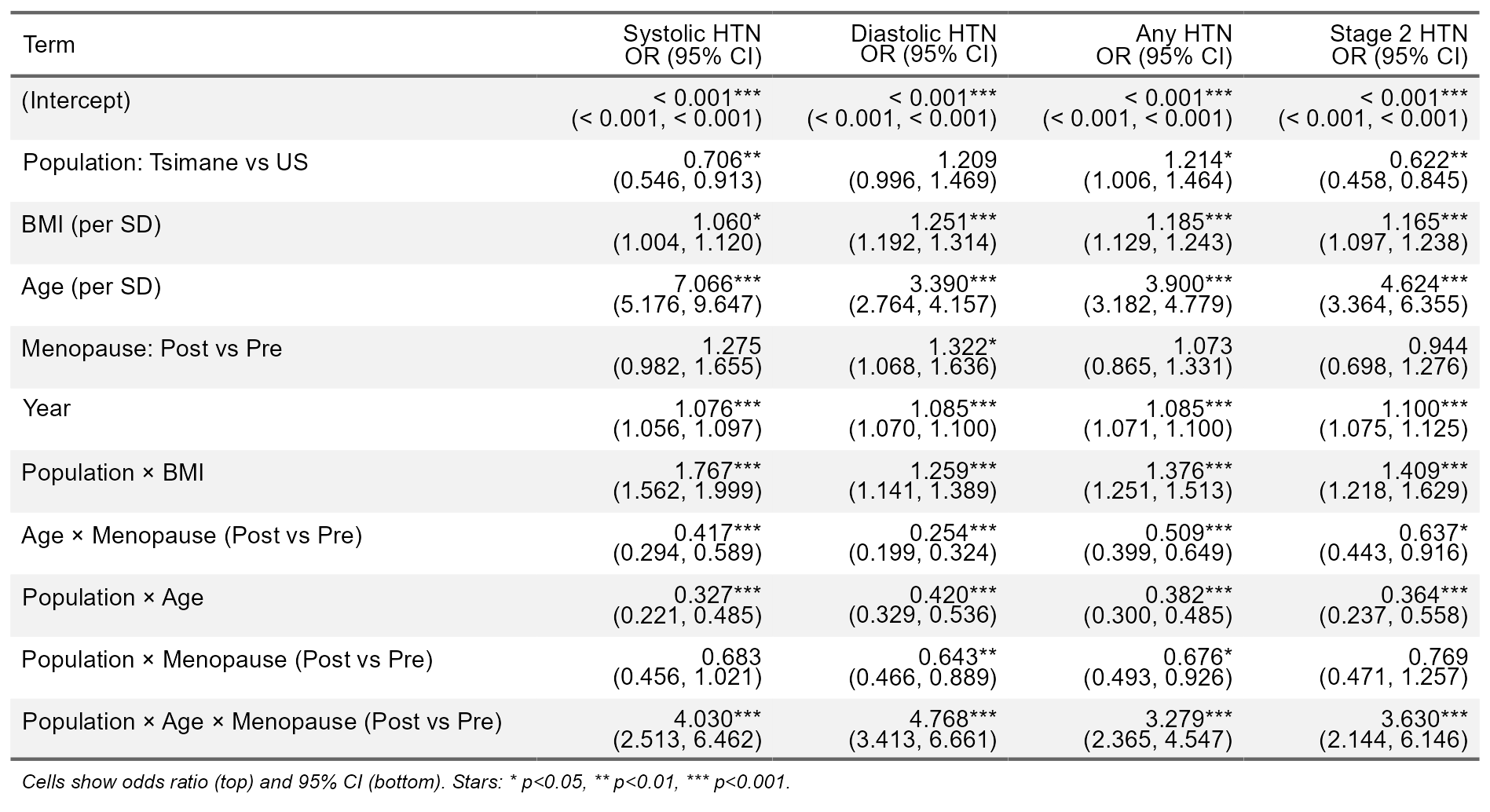
**

**SI Table 3: U.S. and Tsimane Men Logistic Regression Models**

**
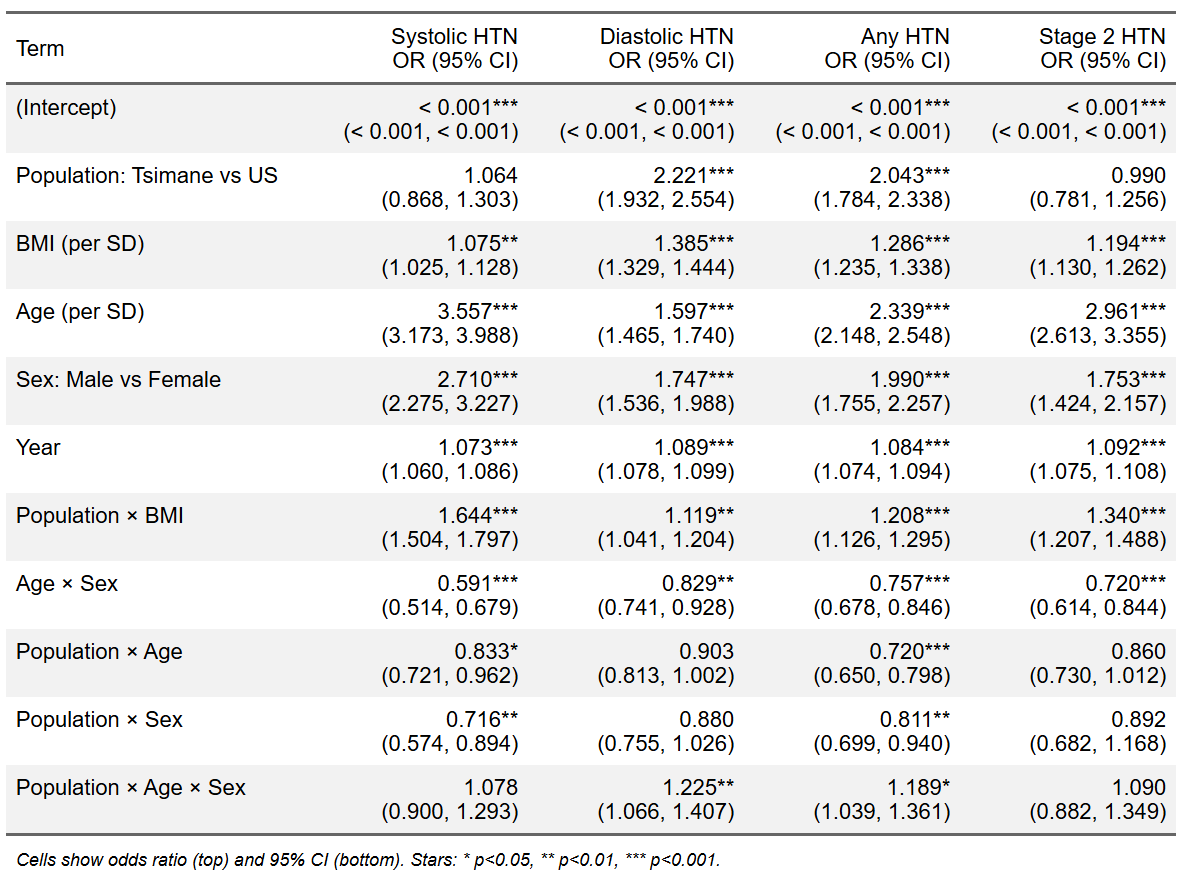
**

**SI Table 4: Tsimane Pre- Versus Post-Menopause Hypertension Incidence Rates**

**
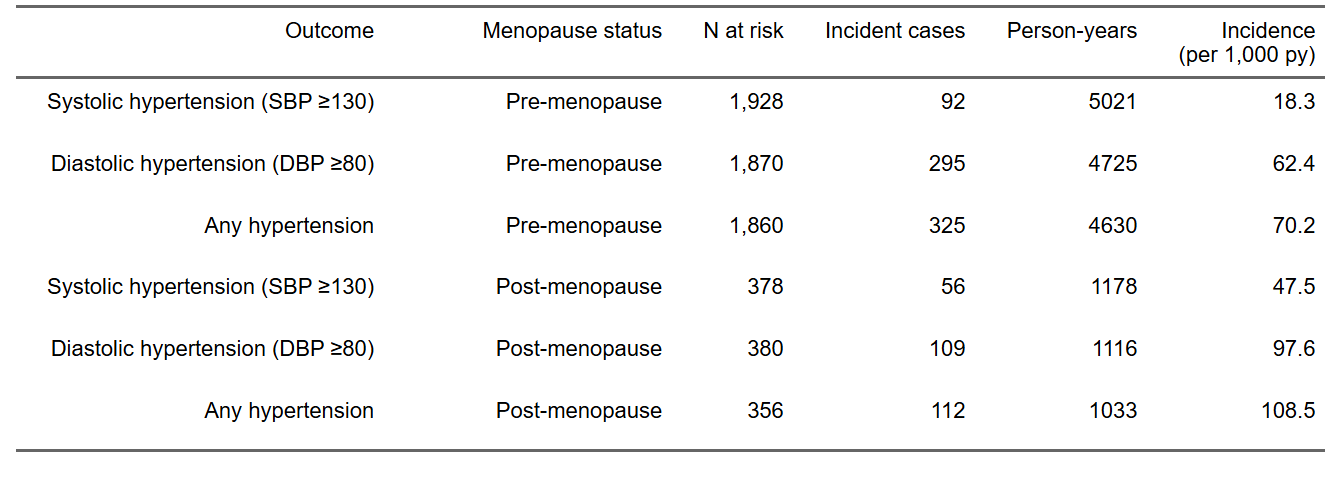
**

**SI Table 5: Linear and Logistic Regression Results Including Tsimane Who Self-Reported Menopause Before Age 40
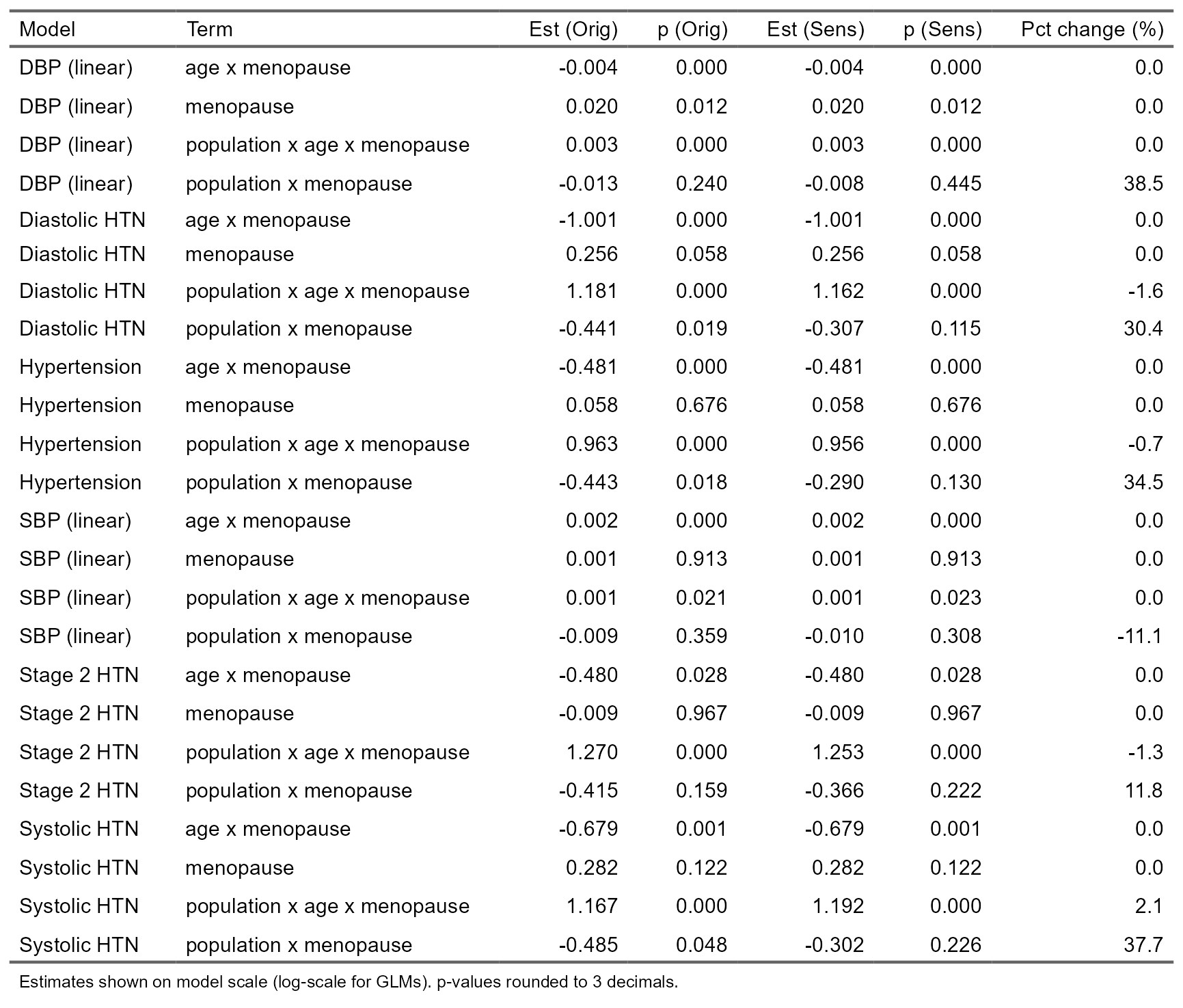
**

**SI Table 6: Estradiol Sensitivity Results Including Tsimane Who Self-Reported Menopause Before Age 40
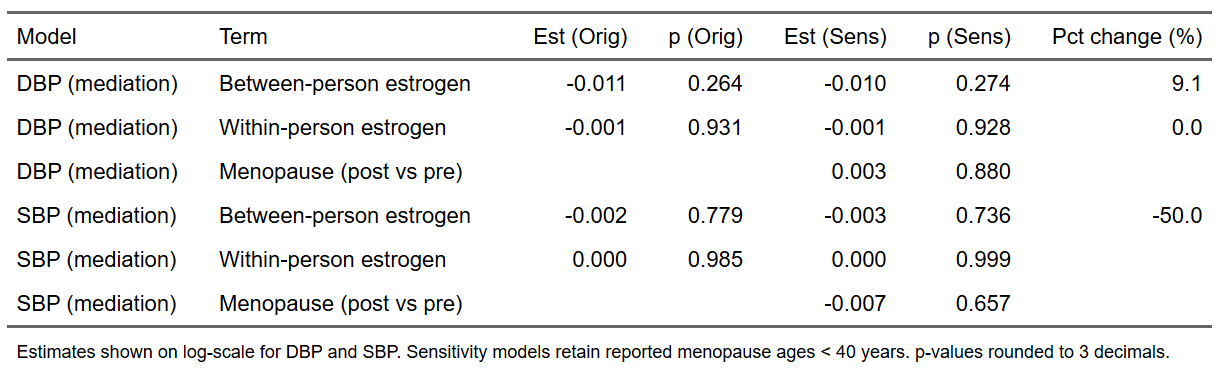
**

**SI Figure 1: Distribution of Tsimane Age at Menopause**

**
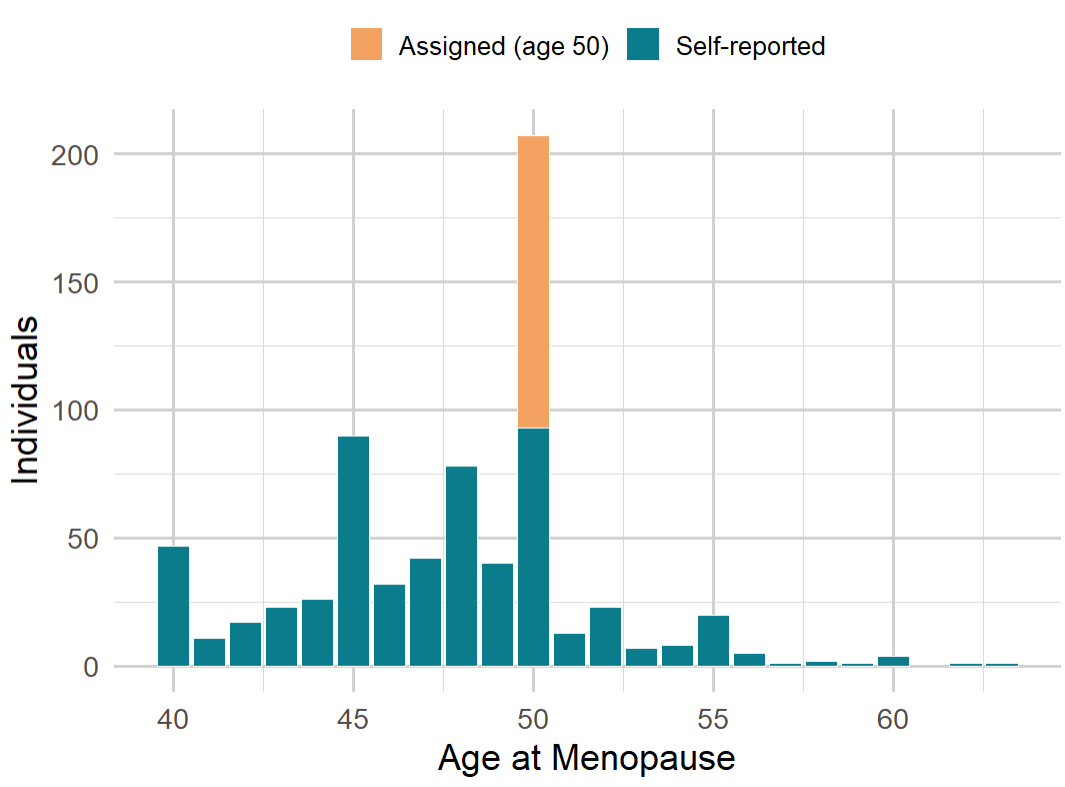
**

**Age at menopause (y) for Tsimane participants. Figure includes women with self-reported reproductive histories (n=608) and women assigned menopause status at age 50 (n=114).**

**SI Figure 2: Tsimane Odds of Hypertension Pre- Versus Post-menopause**

**
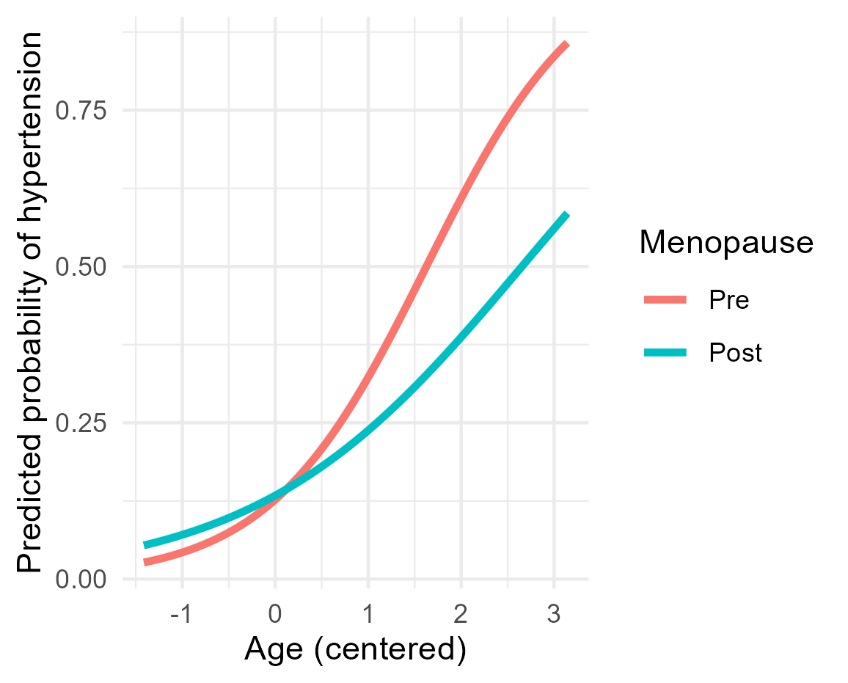
**

**Predicted odds of hypertension as a function of centered age (y) for Tsimane participants. Age is centered at the sample mean. Figure includes women with self-reported reproductive histories (n=608) and women assigned menopause status at age 50 (n=114).**

**SI Figure 3: Tsimane and U.S. SBP, DBP, and Hypertension Prevalence by Year**

**
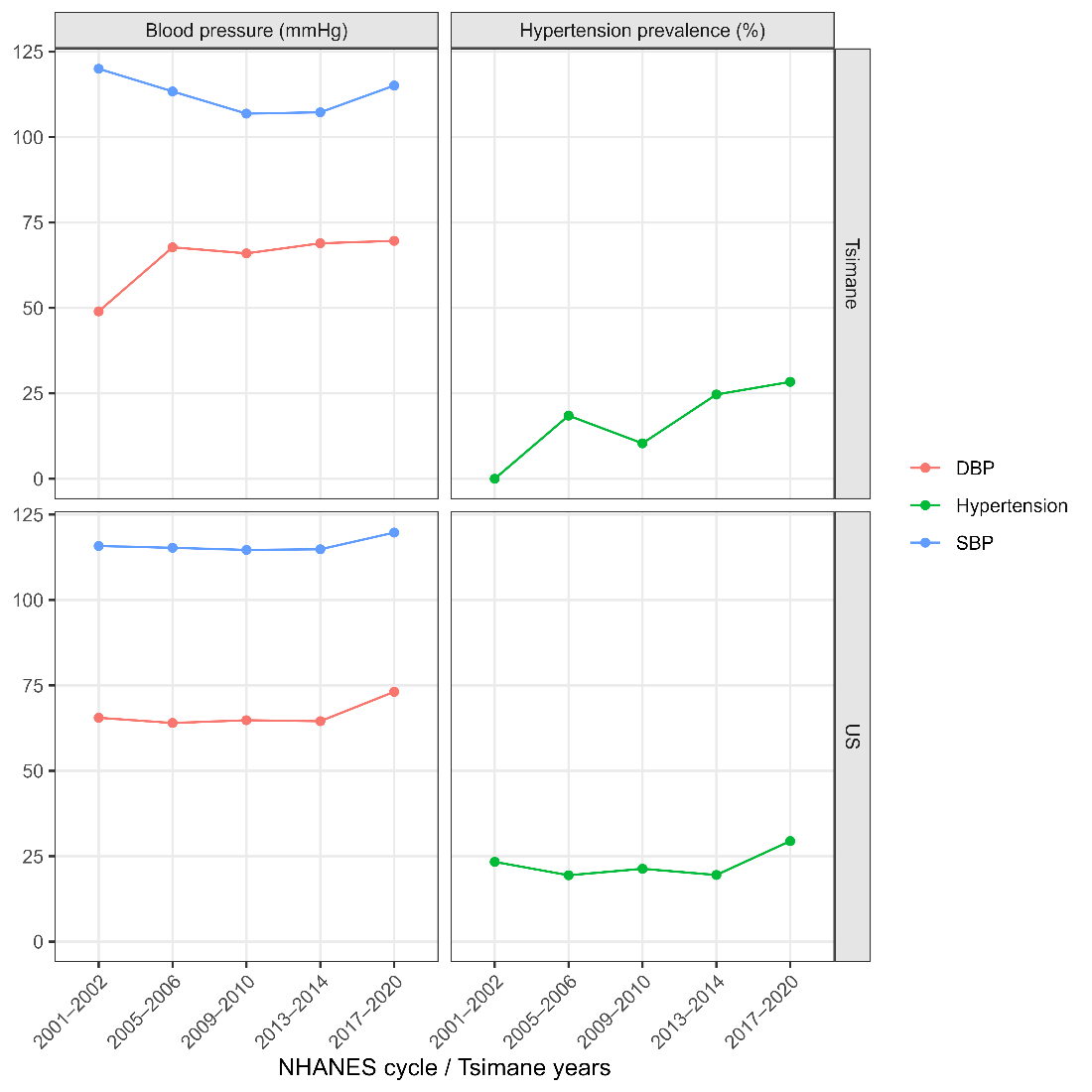
**

**Left: Mean DBP and SBP (mmHg) by Tsimane data collection year or NHANES cycle. Right: Hypertension prevalence (%) by Tsimane data collection year or NHANES cycle. Tsimane data are in the top row and NHANES data are in the bottom row.**
